# Evidence for cumulative cultural evolution in bird song

**DOI:** 10.1098/rstb.2020.0322

**Published:** 2022-01-31

**Authors:** Heather Williams, Robert F. Lachlan

**Affiliations:** ^1^ Biology Department, Williams College, Williamstown, MA 01267, USA; ^2^ Department of Psychology, Royal Holloway University of London, London TW20 0EX, UK

**Keywords:** bird song, categorization, syntax, learning biases, segmentation, cumulative cultural evolution

## Abstract

In studies of cumulative cultural evolution in non-human animals, the focus is most often on incremental changes that increase the efficacy of an existing form of socially learned behaviour, such as the refinement of migratory pathways. In this paper, we compare the songs of different species to describe patterns of evolution in the acoustic structure of bird songs, and explore the question of what building blocks might underlie cumulative cultural evolution of bird song using a comparative approach. We suggest that three steps occurred: first, imitation of independent sounds, or notes, via social learning; second, the formation of categories of note types; and third, assembling note types into sequences with defined structures. Simple sequences can then be repeated to form simple songs or concatenated with other sequences to form segmented songs, increasing complexity. Variant forms of both the notes and the sequencing rules may then arise due to copy errors and innovation. Some variants may become established in the population because of learning biases or selection, increasing signal efficiency, or because of cultural drift. Cumulative cultural evolution of bird songs thus arises from cognitive processes such as vocal imitation, categorization during memorization and learning biases applied to basic acoustic building blocks.

This article is part of a discussion meeting issue ‘The emergence of collective knowledge and cumulative culture in animals, humans and machines’.

## 1. Introduction

Bird songs have many of the characteristics of speech and language: they are learned from conspecific models, consist of sounds that can be divided into distinct categories, and are assembled into sequences that may vary according to syntactic rules on the level of syllables, phrases, songs or bouts [1–6]. As do humpback whale songs [7], these socially learned sounds and sequences evolve as a population's songs change over time [8–13]. This accumulation of changes leads to differentiation of vocal forms across populations [14–16] and to distinct functions for different parts of the repertoire [11,17,18]. As far as we can tell, however, the ‘meanings' encoded in such songs are relatively restricted: we know that singers impart information about their species, as in the Siberian greenish warblers (the *Phylloscopus trochiloides* complex), whose songs differ in frequency and temporal parameters [19]; about population identity, as is the case for white-crowned sparrows (*Zonotrichia leucophrys*) where geographically separate groups each sing a distinct dialect [20]; and about individual identity, as when red-winged blackbird (*Agelaius phoeniceus*) males respond more aggressively to strangers' songs from the same population than to their neighbours' songs [21]. Songs also signal birds' availability as potential mates and their claim to a territory, as demonstrated by studies that remove males and replace them with speakers broadcasting songs [22]. Information about motivation and aggressive intentions is provided by singing the song at lower amplitude [23], overlapping another's song [24], or matching another's song type [25]. The status of the bond between mates is encoded in the coordination of songs of duetting birds [26]. How proficiently specific sections of a song are performed provides cues to a bird's age, status or quality [27–29]. Variation in the syntax of learned vocalizations can encode information about social context [30–32], the probability of movement [33], the presence of food [34] and the presence and type of predators [35]. Nevertheless, learned bird vocalizations appear to be far less complex and have fewer distinctions of meaning than are present in human language.

As for any socially learned behaviour, imprecise transmission introduces variation into bird songs. Within individuals' songs this variation is observable as innovations and copy errors, either in the basic components or in the assembly of those components into sequences. The variants may have different ‘efficiency’, either in terms of communication, or in terms of learnability, or in terms of physiological costs [36]. As is the case for genetic evolution, either drift or selection (or both) may operate to increase or decrease the prevalence of particular learned variants within a population [37,38], resulting in cultural evolution. If this process is repeated, the song forms used by a population may continue to change and evolve, but successive changes alone are not cumulative cultural evolution. The criteria for cumulative cultural evolution [39] are satisfied when (a) changes in a population's song accumulate in succession and (b) the new features that are adopted at different time points increase the complexity and efficiency of the song.

Many songbird species learn their songs during a restricted critical period early in life, singing a crystallized song thereafter [40–43]. Because learning is not continuous, critical period learners allow us to distinguish between the social learning processes that underlie cultural evolution and the consequences for the adult of learning a specific form of the behaviour. In this review, we look at how song learning generates variation within acoustic space, how acoustic categories are formed, and how those categories serve as building blocks that are assembled into simple sequences. We suggest that the resulting simple sequences, or syllables, in turn, serve as building blocks that are concatenated into songs with two levels of syntactic structure: syllables and segments. In songs with multiple segments, each segment may then come to play a different signalling function because of different cultural evolution mechanisms.

## 2. Song learning, variation and category formation

The developmental stages in the learning of an adult song have been described as (a) memorization, (b) calibrating the vocal organ (subsong) and producing precursor syllables and (c) imitating the memorized sounds (plastic song) [44]. Variation can appear at any stage: as the result of inaccurate storage in memory, because novel sounds arise during the vocal calibration process that then persist through other stages, or as a consequence of inaccurately reproducing the memorized version during plastic song. All of these processes can be considered as introducing ‘noise’ into the representation of the model for the song, resulting in the use of an expanded acoustic space during the learning process. This variation or noise may or may not be discarded or reconfigured as learning proceeds. Many birds produce more notes, syllables, phrases and songs^1^ than they include in their adult repertoire [45,46], sometimes because they produce more songs than they need during development and sometimes as result of innovations they generate. Which song material is retained by an adult can be influenced by what a bird hears late in the song learning process [42].

The mature birdsong of critical period learners is said to be ‘crystallized’; the components (notes and syllables) are produced in a stereotyped manner that is consistent across renditions [47]. Within a population, there may typically be hundreds (and frequently thousands) of different song types. However, the notes and syllables that make up a population's songs often fall into clusters: the ‘downsweeps' and ‘stacks' of zebra finches (*Taenopygia guttata*) [48,49], the distinct note categories of swamp sparrows (*Melospiza georgiana*) [50]*,* the distinctive middle segment notes of Savannah sparrows (*Passerculus sandwichensis*) [11]*.* These clusters are, to a greater or lesser extent, separated in acoustic space by characteristics such as frequency, bandwidth, modulation and duration (analogous to the acoustic features that define vowels in human speech) [51]. In the case of human speech, the emergence of categories within acoustic space has been modelled using several approaches, with the outcome defined as ‘distinguishability’ either in perceptual space (how distinct the categories are to the hearer) or in the motor space used to produce speech sounds (how distinct the articulatory gestures are to the producer) [52–54]. When learning and production cycles are iterated, and outputs are retained in the population on the basis of distinguishability, categories of speech sounds such as vowels emerge. However, these approaches to cultural evolution of speech most often either implicitly or explicitly include the assumption that distinct categories map onto separate meanings, and that the functional demands of the signalling system—the need to encode more meanings—drive the addition of more categories to the acoustic space used in the vocalizations. In the case of bird songs, there does not appear to be a strong selective pressure to define additional syllable categories or flexible sequences that are associated with distinct meanings. Nevertheless, bird songs have clearly defined note categories, as well as rules for assembling those categories into higher-order structure. How might such syllable categories and sequences arise in the absence of evolutionary pressure for expanded meaning?

**Figure 1.**
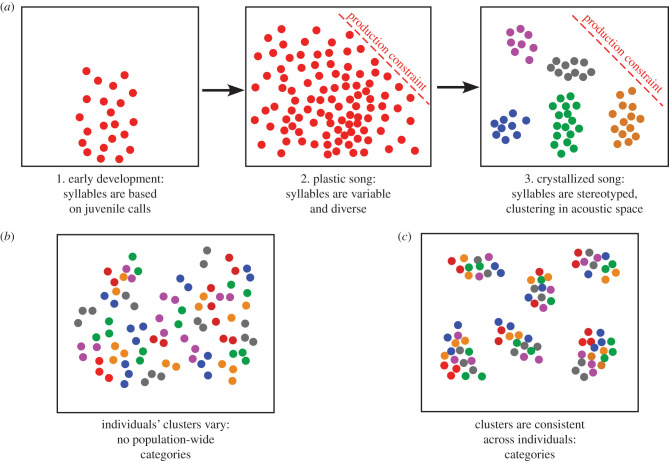
Formation of syllable categories during development. (*a*) Schematic showing use of acoustic space during song development. Primordial syllables are similar to juvenile calls (often food begging calls). As song learning progresses, the sounds produced diversify and occupy a larger acoustic space, although production constraints may limit which sounds can be sung. As learning proceeds, notes converge and become more stereotyped, forming clusters within the acoustic space. (*b*) When each individual (denoted by a different colour) sings syllables that fall within narrow regions of acoustic space, and different individuals use different parts of that acoustic space, there are no distinct population-wide categories. (*c*) Population-wide categories arise when the syllable clusters of different individuals coincide in acoustic space.

One possible mechanism for the formation of distinct acoustic categories is a combination of noise and learning biases. Noise in the memorization and learning process could first expand the acoustic space a bird uses during vocal learning, and learning biases would then define what is retained and what is discarded as the song matures. For example, in zebra finches the initial units of developing songs are nearly identical and similar to noisy begging calls [55]. These units diverge as song learning progresses, guided by memories of the model's song and becoming less noisy and more acoustically defined in the process [51]. Schematically, the trajectory of song learning thus starts with sounds occupying a broad acoustic space. As learning progresses note types emerge, forming clusters, or categories, within that space (figure 1*a*). In this scenario, each individual's note types might be idiosyncratic. If the note types of several individuals produce overlapping categories, a population's syllables, as a whole, could occupy most of the acoustic space (figure 1*b*), and there would be no distinct acoustic categories within the songs of a population. Alternatively, the notes of all the birds in a population might converge upon the same subsets of acoustic space (figure 1*c*), forming distinct, population-wide acoustic categories.

In some species, there are innate predispositions for learning sounds that occupy distinct subregions of the acoustic space. Peter Marler's work was the first to investigate such species-specific acoustic categories in detail: swamp sparrows tutored with both swamp and song sparrow (*Melospiza melodia*) syllables include only swamp sparrow syllables in their own songs, regardless of whether those syllables are arranged in species-typical syntax within the tutor songs [56,57]. A bias towards learning the sounds sung by one's own species is also present in other songbirds, such as white-crowned sparrows [58,59]*.* Birds raised hearing untutored, depauperate songs of their own species [60,61]—or even hearing only recordings of their own developing vocalizations [62]—sing syllables and songs that are closer to the species-typical versions sung by normally reared birds. These results raise the possibility that population-wide categories are the consequence of ‘lumpiness' in the acoustic space that a species uses, with birds being genetically predisposed to learn certain subsets or ranges of acoustic features. When categories form they will lie within these species-specific subsets of the possible acoustic space.

Biases in song learning might also be the result of articulatory constraints. Anatomical or neuromuscular constraints on articulatory gestures may delimit the acoustic space a bird uses. For example, birds with larger, more massive beaks tend to produce smaller frequency ranges within a single sound, as the acoustic filtering provided by changes in beak aperture cannot change as quickly as in birds with smaller beaks [63–65]. Another form of articulatory categorization arises from the fact that songbirds have a two-part syrinx. Each syringeal half is associated with one bronchus, giving the entire syrinx two independent ‘voices’ [66,67]. The right syringeal half produces high-frequency sounds, while the left side produces low-frequency sounds. This partitioning of frequencies between the two sides of the vocal organ is consistent across a number of species, including canaries (*Serinus canaria*) [68,69]*,* zebra finches [70] and brown-headed cowbirds (*Molothrus ater*) [71]. Specialization for different frequency ranges in the two sides of the syrinx is also present in oilbirds (*Steatornis caripensis*) [72]*,* which are not songbirds and do not learn their vocalizations, suggesting that the distinctive outputs of the two sides of the vocal organ might form a physiological basis for the initial emergence of sound categories.

The examples above describe content-based biases in cultural transmission. Frequency-based learning biases, specifically conformity, guide song development in some songbird species. Lachlan *et al*. [73] compared present-day distributions of syllable types across six swamp sparrow populations with the outcomes of simulations that used different learning biases and parameters. In the absence of a learning bias, neither a low nor a high mutation/innovation rate yielded a good match to field data. A conformist bias resulted in a much better fit to the data than did a demonstrator or a content bias, suggesting that swamp sparrows preferentially learn syllables they have heard sung by more tutors. Such a pattern of transmission tends to remove rare variants from a population and so gives rise to clustered patterns of variation. In such cases, the location of clusters in acoustic space may be arbitrary.

**Figure 2.**
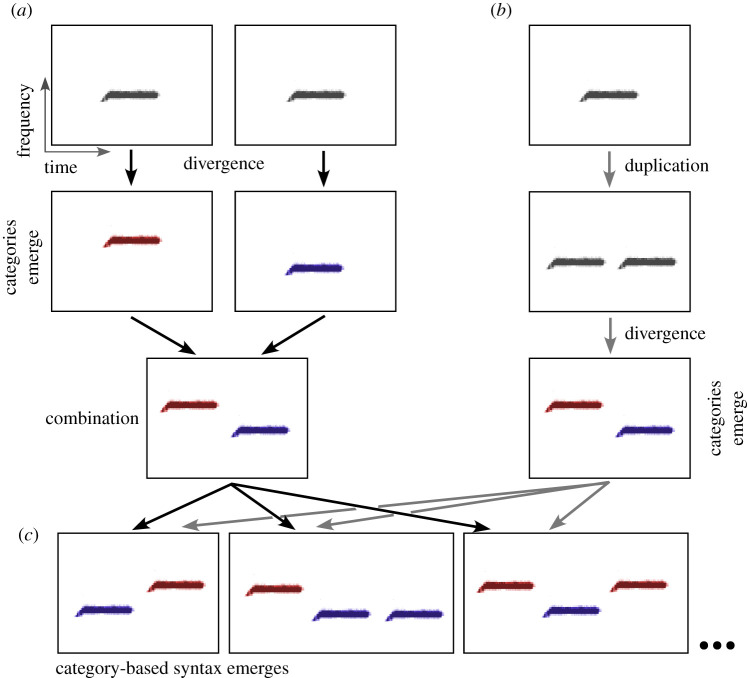
Two pathways to category-based syntax. (*a*) Divergence: notes sung by birds from separate lineages diverge to form different types. When an individual learns and sings both types, an ordered sequence arises. (*b*) Duplication: a single bird initially sings two duplicate notes, which then diverge from the original form, forming an ordered sequence of two note types. (*c*) In either case, further sequences arise as inaccurate copying or innovation changes the order or introduces additional copies into the sequence.

We propose that clusters, or categories, are a building block for the cultural evolution of syntactic structure. Consider a species singing a single learned note type as its vocalization. Two possibilities for the emergence of distinct categories exist: (1) two birds might produce different note types or (2) a single bird could produce more than one note type. Learning, with its attendant variation in the form of noise, copying errors and innovation, makes the first scenario possible: different lineages could produce variations on a single sound (perhaps as simply as one lineage producing a version that emphasizes the output of the left side of the syrinx and another emphasizing a higher frequency produced with the right side). These two variants, diverging over time, could come to distinguish the two lineages (figure 2*a*). At that point, a bird that by chance learned and sang notes from two different lineages could produce a simple ordered sequence. Alternatively, duplication—repeating a single sound during learning or production—could initially result in a lineage that produces two identical notes (figure 2*b*). The acoustic characteristics of the two sounds could then diverge within that two-note vocalization. Duplication followed by divergence would result in not only two categories of sounds but also, simultaneously, in a simple ordered sequence. Once ordered sequences exist, they may be elaborated, either by additional cycles of duplication and divergence, or by learning sequences from more than one tutor and concatenating or mixing them (figure 2*c*). Either scenario gives rise to the rudiments of syntax.

## 3. Note categories and syllable syntax in swamp sparrow songs

**Figure 3.**
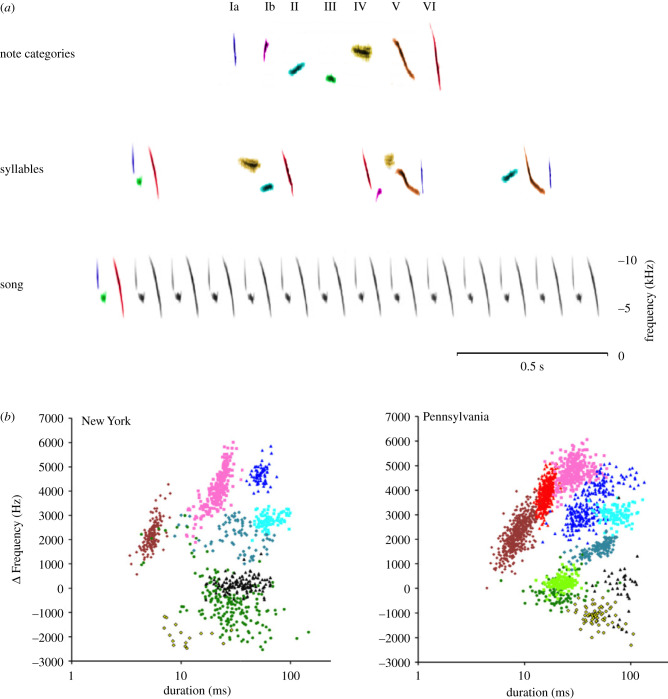
Swamp sparrow note categories and syllables. (*a*) Marler & Pickert [50] originally defined six categories; sub-categories Ia and Ib were split by Lachlan *et al*. [74] based on cluster analyses of acoustic characteristics. Syllables are assembled from 2 to 5 note categories, and songs are formed by repeating a single syllable (individual birds sing 2–5 different songs [74]). (*b*) Individual notes can be characterized largely by their duration and change in frequency. When notes are clustered using this approach using Gaussian mixture models, populations in New York and Pennsylvania differ in number of clusters and where those clusters are located in acoustic space. After Lachlan *et al.* [73,74].

The most complete description of how note categories form and are assembled into higher-order structures within bird songs comes from swamp sparrows. In this species, notes are nearly always simple uninflected frequency sweeps, making it relatively easy to characterize differences between them. Within each population there are a limited number of note clusters: six categories were originally defined by visual scoring of sonograms from a New York population [50], while statistical clustering analyses found evidence for seven or eight categories (figure 3*a*) [74,75]. While clearly defined within each population, clusters vary between populations in their number and their location in acoustic space [75] (figure 1*a*). The variation between populations suggests that swamp sparrow note type clusters are not based on underlying innate categories, but emerge from cultural evolutionary processes.

To build syllables from notes, swamp sparrows assemble 2–5 notes into a short sequence (figure 3*a*) [50]. These syllables are then repeated 10–15 times to form songs. Although the algorithm for generating songs from syllables is thus both simple and universal, the rules for assembling notes into syllables can vary across populations. Notes from a given category tend to be used consistently at specific positions within a syllable: for example, in a New York population, short category I notes usually occur only at the beginning of syllables, while longer category VI notes are primarily sung in the final position within syllables [50,74,76]. However, in a Pennsylvania population, there is an additional intermediate-length cluster that can be found either at the start or the end of the syllable [75], while in other populations, the order is reversed [76]. The overarching syntactical rule for generating syllables—concatenate 2–5 notes drawn from different categories—is species-specific, but the role each constituent note category can play within that structure varies across populations, in a manner reminiscent of human languages.

Swamp sparrow note categories and the assembly of those categories into syllables have both perceptual and motor correlates. In field playback studies, New York birds perceived note types I and VI, which differ in duration, as two distinct categories [77], even though the variation in the stimulus note duration was continuous. When the experiment was repeated in Pennsylvania, the perceptual boundaries varied depending on the position of the note: at the beginning of syllables, short (type I) and intermediate types were perceived as part of the same category, while at the end of the syllable, long (type VI) and intermediate types were perceived as part of the same category. The perceptual boundaries found in behavioural studies were consistent with those described in parallel electrophysiological studies [78].

Together, these results suggest a hierarchical organization of learning. Social learning of note types first led to the cultural evolution of population-specific clusters. Once these clusters were established, we suggest that birds represented these note clusters as perceptual categories, making sense of regularities in their auditory environment, much as infant humans learn phonemic categories [79]. This categorization of notes then fed back into learning, further separating and defining note type clusters. Then, as some syllable types became more common than others within the population, it provided birds with an additional regularity: the sequence of note categories within the syllable. These ‘phonemic rules' were then learned as well. Critically, these different aspects of learning appear to build upon each other, sometimes adding complexity to birds' knowledge of their population's songs, even when the songs themselves did not become more complex.

**Figure 4.**
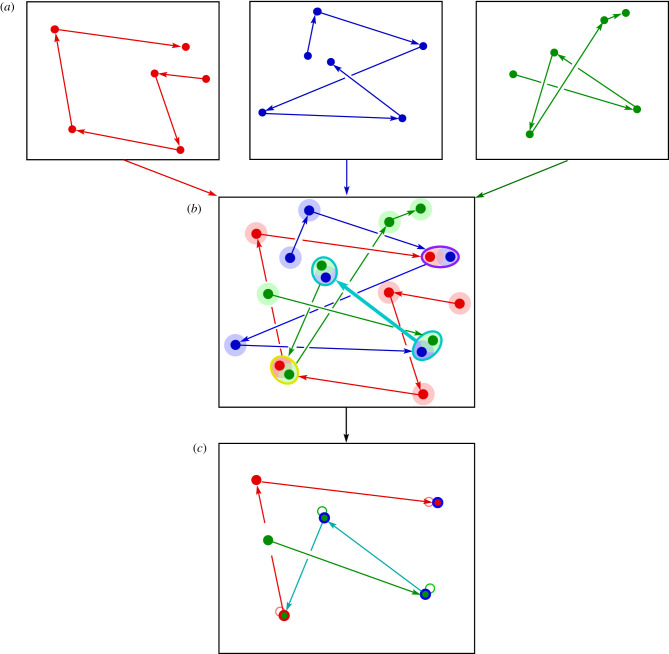
Categories and organized syntax emerge from random note sequences. (*a*) In this schematic, a young bird hears and memorizes three adult songs (red, green and blue), represented in as random strings of six notes. Each note is represented as a point in a two-dimensional acoustic space. (*b*) Notes with overlapping characteristics combine to form a single representation, or category (circled pairs of notes), as do shared transitions between two note categories (thicker turquoise arrow). If learners use conformist bias, note categories that include more than one memorized example are more likely to persist in the adult song, as are transitions between such categories. (*c*) In this simple example, the young bird's final song includes four syllables from ‘categories' present in more than one model song, connected by transitions between syllables that were present in the model songs. The first four syllables follow the sequence of the original green song (and, in part, the blue song). At the fourth syllable, the order switches to that of the last three syllables of the original red song. In a population of learners that sample many of the same songs and use a simple conformist learning bias, note categories and a systematic syntax will emerge over time.

In this scenario, cultural transmission of syllables favours the emergence of note type clusters. Increasing sophistication of swamp sparrow perception, such as conformist biases, would shape the cultural transmission of swamp sparrow song, resulting in a relatively small number of syllable types becoming very common [73], thereby favouring the establishment of note type clusters. This conformist bias may be related to the fact that swamp sparrows recognize and respond most strongly to more ‘typical’ versions of syllables—those that are centrally located in the acoustic space occupied by a syllable type [80]. In field playbacks, males respond more aggressively to typical syllables, and in the laboratory, females primed with oestradiol give more copulation solicitation displays to typical syllables. This finding has been connected to the developmental stress hypothesis, the idea that receivers might assess singers based on how well they were able to learn their songs [81]. Interestingly, a theoretical model found that in order for learning accuracy to serve as a marker of developmental stress, both receivers and signallers need to categorize the songs that they hear early in life [82]. The process of categorization might thus form the basis for the evolution and refinement of units carrying information about fitness within a communication signal. However, as far as we know, neither the different note categories nor the different syllables formed by combinatorial assembly of note categories carry different referential meanings. Note categories and syntactic rules within learned communication systems need not be predicated on the need to generate more signals to encode more meanings—simple learning biases alone may be sufficient to drive the evolution of structure, as is schematically illustrated in figure 4.

## 4. Interactions between the formation of note categories and higher-level structure

As we observe them now, 50 Myr after passerine songbirds first emerged, one prominent characteristic of bird songs is their species-characteristic note categories and syntax. For example, chaffinches (*Fringilla coelebs*) [83,84] and white-crowned sparrows [85] sing distinctive whistles, trills and note combinations at the beginnings and ends of their songs, zebra finches sing longer syllables at the ends of each repeated motif [86], northern mockingbirds (*Mimus polyglottos*) repeat each phrase three times [87], and Savannah sparrows start each song with an accelerating sequence of high-frequency downsweeps [88]. Studies of the development of deaf birds and of birds raised in isolation find that songs with aspects of species-specific sequencing develop in the absence of a model, and so we know that some elements of a species' normal syntax are not learned [89–92]. These unlearned components of syntax could be based upon perceptual predispositions, motor biases favouring the chaining of particular articulatory gestures, or both. The idea that syllable categories and syntactic rules interact is further supported by work on song development. Young zebra finches use two distinct song learning strategies: (a) simultaneous development of the phonology and sequence of notes or (b) first producing repeated identical syllables, some of which are modified and arranged to form a sequence [93]. Individual birds may use either strategy, or may combine the two strategies. When zebra finches are challenged to learn new material late in song development they first alter existing syllables to match models' phonology, and then attempt to adjust the order of those syllables [94]. Crystallized canary songs consist of a series of phrases, with each phrase made up of a string of identical syllables [95,96]. When young canaries were tutored with ‘glissando’ songs that consisted of a long series of otherwise identical syllables that varied in frequency, forming a long, smooth transition from high to low, they initially copied these glissandos accurately [97]. However, as song learning progressed, species-specific phrase structure and categories emerged: the glissando string was broken up into phrases, and each phrase became a short repeated string of one syllable drawn from the original continuum of notes. Over the course of song learning, these canaries first accurately copied an atypical song, and then altered it by reducing a continuous series of note types to categories, and repeating each category of note to form species-typical phrases.

As song learning studies illustrate, note and syllable categories do not develop completely independently. A single utterance may be repeated, giving rise to a string of initially identical notes or syllables that differentiate to form a sequence. Alternatively, a continuum of notes may converge to form a single type. Two different sounds can also be integrated during development to form a note: northern cardinals (*Cardinalis cardinalis*) produce long descending or ascending notes that span a broad frequency range and appear to be a single syllable but are, in motor terms, two separate vocalizations, each produced by one half of the syrinx [98]. During development, birds must learn to combine these two separate notes so that they become a single continuous sound. We do not know whether these developmental trajectories parallel the evolution of note categories and the rules used for assembling them. If note types or categories evolved first and sequences emerged later, the seemingly simultaneous emergence of note categories and syntax during development (as seems to be the case in canaries) would be a later adaptation, perhaps to increase the efficiency of learning. Alternatively, song development might recapitulate evolution, with ordered sequences emerging after initially identical repeated notes differentiate into distinct syllables. In either scenario, innate specification of acoustic parameters must combine with social learning to generate the basic building blocks that are then assembled, using structural rules that also have innate and learned components, into songs that are species-specific yet vary across populations and individuals.

## 5. Complexity

For the purpose of comparing vocal communication systems, we use the term ‘complexity’ to describe the number of different sequences that can be generated; complexity increases with the number of sound categories and the number of combinations that syntactic rules can generate from those sounds. Research using iterated learning of artificial languages suggests that selection for learnability favours systems that are easy to learn but are impoverished in the ability to communicate information [99]. In contrast, selection for the ability to communicate many different meanings favours languages that have distinguishable components that carry different information, but may be hard to learn, while selection for both learnability and communication favours the evolution of languages that are compositional, using combinatorial rules to assemble learnable sound units into different sequences [100]. Like natural human languages, bird songs appear to be under selection for learnability, which may be related to morphological or physiological constraints [101,102] as well as to cognitive mechanisms [73,80]. Although, as we have noted, the information communicated by bird songs is far less rich than for natural human languages, multiple note categories that are assembled into structured sequences generate enough complexity to signal a variety of meanings.

**Figure 5.**
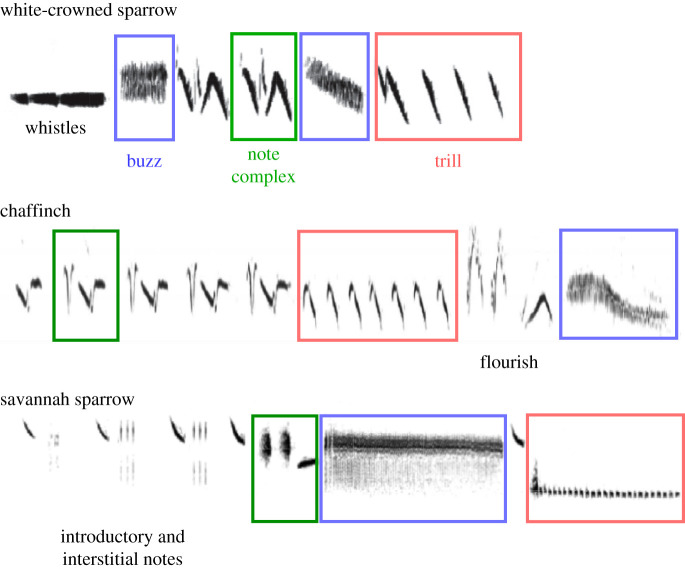
Segmented song structure, as exemplified by white crowned sparrows, chaffinches and Savannah sparrows. Segments may be defined as: note complexes similar to the syllables of swamp sparrows (green), buzzes consisting of very short broadband notes repeated in rapid succession (blue) and trills made up of a repeated sequence of longer, more tonal notes (red). So that structure can be better compared across the three species' songs, the time (*x*-axis) and frequency scales (*y*-axis) for the three songs shown here are similar but not identical (white crowned-sparrow song = 2.5 s; chaffinch song = 3 s; Savannah sparrow song = 2.5 s). Spectrograms from Nelson [18], Lachlan *et al.* [74] and Williams *et al.* [112].

Songbirds do use simple combinatorial communication systems. Some bird calls (calls are defined as relatively short vocalizations that are not used in courtship or territory defence), such as those of the Paridae [34,103–106] and Australian babblers [107], appear to have a simple combinatorial syntax that signals different meanings in a way that is analogous to human speech. However, the number of different call types and combinations in these systems is relatively small, comparable to the notes and syllables of swamp sparrows. Other species' songs are quite complex: individual birds' repertoires may include dozens of note types or hundreds of different sequences (e.g. grey catbirds, *Dumetella carolinensis* [108]; winter wrens, *Troglodytes troglodytes* [109]; common nightingales, *Luscinia megarhynchos* [110]). Still, other species vary the way songs are sung within a bout, yielding a further layer of sequence complexity [6,111]. In this section, we focus on an intermediate level of song complexity: continuous sequences that last 2–4 s and include at least two segments, each with its own set of notes and structure. Some examples are the songs of Savannah sparrows, chaffinches and white-crowned sparrows (figure 5); they are more amenable to analysis than large repertoires or very long songs. White-crowned sparrow songs begin with one or more whistles, followed by one or more note complexes, and conclude with a trill [85]. Chaffinch songs begin with one or more repeated note complexes or trills and conclude with a flourish that typically includes a terminal buzz [74]. Savannah sparrow songs begin with a series of introductory notes and associated interstitial notes, followed by a note complex, a buzz and a trill [43]. These songs share the characteristics of being composed of distinct song segments, each consisting of one or more note types organized according to segment-specific rules.

Segmented song structures can be represented as a string of concatenated simpler structures. Each of the segments within these songs is similar in complexity to swamp sparrow songs: one or more notes are assembled into a syllable that may or may not be repeated to form the segment. Two to six segments are then assembled to form the song. As do the note types, segment types fall into categories (buzzes, trills and note complexes), and those categories play consistent roles in the structure of a species' song, in a manner parallel to the role of notes in forming swamp sparrow syllables. A segmented structure can be generated by iterations in which building blocks are assembled into sequences: (1) notes are the building blocks for syllables, (2) syllables can be repeated to form segments and (3) segments are the building blocks for songs. The rules for assembling these structures may have initially arisen so as to allow individuals to learn efficiently (for example, categorization of notes facilitates memorization) and/or to present the components of an individual's vocal repertoire to its hearers efficiently, so that all of an individual's learned notes can be heard within a single vocalization that is organized both to emphasize the characteristics of those notes and to allow for easy comparison of multiple songs. In such songs, notes, syllables, segments and songs may not carry meaning, but instead may form the basis for hearers to assess the accuracy of learning and the size and variety of the repertoire that was learned.

As we have seen, learning biases, natural and sexual selection, and random factors such as population size and drift can result in cultural evolution of a population's song in terms of notes, syllables and structure [113,114]. In some species, separate song types may serve different functions and so evolve in different directions and at different rates [17,115]. When songs are formed of distinct concatenated segments, cultural evolution can act differently on individual segments, and the functions of segments may diverge [116]. For example, male red-winged blackbirds sing a two-segment song; females respond more strongly when the initial whistled segment was present, while the long trill that forms the second part of the song is sufficient to elicit strong male responses [117].

Many songbird species have learned song features that are population markers: segments that are shared by individuals within a population and so define a local dialect. The white-crowned sparrow's terminal trill [18,118] and the Savannah sparrow's buzz [112] are consistent across most or all individuals within a population. Modelling studies suggest several factors and mechanisms that might be responsible for maintenance of dialects [119–127], including drift, philopatry, learning after dispersal, population turnover and conformist learning biases. Playback studies in a number of species [112,128–130] find that males respond more strongly not only to the local population's song but also specifically to song segments that define a dialect or are characteristic of a population.

Other song segments—often note complexes, such as those in the songs of white-crowned and Savannah sparrows—vary substantially between individuals within a population and may serve in part to denote individual or small-group identity [11,18]. Such variability might arise either randomly or because of a relaxation of learning constraints similar to that seen in small or isolated populations [131,132], and the new forms might become established through some combination of (a) cultural drift [37,133] and (b) rare-form bias (anti-conformity) [134].

**Figure 6.**
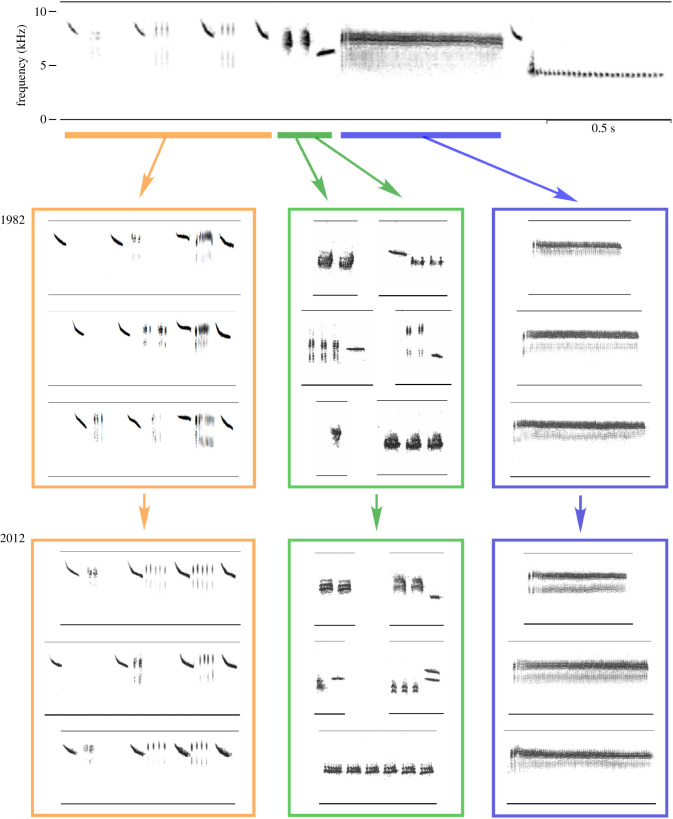
Cultural evolution differs across Savannah sparrow song segments. A full Kent Island song is shown at the top of the figure; below are examples of song segments recorded from the same population 30 years apart. The buzz (blue) varied in length across individuals but was otherwise similar within the population, and the buzzes recorded at the beginning and end of the 30-year interval were statistically identical. The note complex (green) varied both across individuals and across years, although the structure and many of the notes were consistent over time within the population. The introductory section (orange) consists of several repeated downsweeps that do not vary substantially across populations. The softer interstitial notes that fall between the introductory notes were consistent within the population in both 1982 and 2012 but changed over time. In 1982 that pattern was a three-part cluster consisting of short repeated notes/an unmodulated high note/short high trill. It was replaced over the succeeding decades by a series of clicks, as seen in the 2012 examples. The replacement was gradual, and both of the interstitial note patterns were sung in the 1990s and early 2000s.

Because different song segments may serve different signalling functions, they may also be shaped by different learning mechanisms or biases. For example, the Savannah sparrow buzz segment serves as a population marker and is maintained across individuals and time (figure 6, blue) [112], while the middle segment's note complex varies both within a population and over time and appears to denote individual identity or local affiliation (figure 6, green) [11]. The fact that birds apply different learning rules to different song segments adds a level of cognitive complexity to the cultural evolution of bird song.

Another form of signal effectiveness that cultural evolution acts upon is the ability of songs to attract females. An attractive songs yields an increase in the singer's reproductive rate, a standard measure of ‘fitness' in terms of both natural and sexual selection. If one song segment is attractive to females, singing that segment gives a male clear fitness benefits. Cultural evolution may then act on the sexually selected song segment if males are able to preferentially learn variants they observe to be more effective for attracting females (a form of prestige bias). The interstitial notes in the introductory segment of the songs in one Savannah sparrow population (figure 6, orange) changed from one form, high note clusters, to another, click trains, between 1982 and 2011 [11]. During an intermediate period when both forms were sung within the population, males singing the new click train form fledged more young from their nests, suggesting that the novel form was a more effective signal because of a sexual selection advantage. It is not clear why this advantage existed. Perhaps the new version was favoured by a female sensory bias; perhaps females learn their preferences, as in mate choice copying by guppies (*Poecilia reticulata*) [135], and so those preferences are themselves subject to cultural evolution.

These observations highlight several important points about the segments of relatively short learned songs. The segments are (a) made up of different note types organized according to different structural rules, (b) may carry different information, (c) may evolve in different directions and (d) may be shaped by different mechanisms.

## 6. Cumulative cultural evolution: iterated change, effectiveness and complexity

Definitions of cumulative cultural evolution all call for the presence of successive changes in a socially learned behaviour. To meet this criterion, a population's behaviour must have distinctly different forms at distinctly different time periods, perhaps separated by years or generations. At least one additional criterion must also be satisfied, usually defined as either (a) increased behavioural complexity/elaboration [136,137] or (b) increased efficiency in terms of relative costs and benefits [36,39]. Increased complexity (or elaboration) may occur as additional steps in a chain of behaviours, or, in the case of vocalizations, as a more elaborate syntax or an increased number of sound categories. Increased efficiency may take the form of making the behaviour easier to perform (thus reducing physiological costs), easier to learn (reducing developmental costs), or it may make the behaviour itself more efficient: in the case of a communication system, the signals could carry more meanings or provide a more effective signal that better conveys a meaning. We argue that songs based on rules for assembling categories (such as those of swamp sparrows) as well as segmented songs (such as those of Savannah sparrows and white-crowned sparrows) meet these criteria for cumulative cultural evolution. First, iterations of social learning across generations result in systematic changes in a population's songs over time. Second, the successive changes can result in a more effective signal, in terms of reproductive success, as in the case of the interstitial notes of Savannah sparrow song. Third, these songs have evolved at least two layers of organization—categories and syllables for swamp sparrow songs, and syllables and segments for Savannah sparrow song—and so have become more complex and structured over time.

As signalling systems, however, bird songs do not come close to the complexity and combinatoriality of human language, and they also appear to lack referential properties. In many songbird species, learning is completed relatively early in life and new material is not incorporated thereafter, and so the ability of the signal to change during an individual's adult life in order to carry new information is further limited. Nevertheless, variation in the components of the song, the syntax of the song, the performance the song, and the repertoire size carries information about the singer's identity, quality and motivation. Note categories and sequencing rules that are universal within a species' song are likely to have a genetically defined component. Because of these innate predispositions and because individuals within populations that hear each other during development are likely to learn from each other and to share vocalizations, birds within a population often sing a common dialect. These factors would seem to act in concert to homogenize the learned songs within a population. However, copying errors and innovation (and perhaps anti-conformity) lead to the production of novel song features, which provide cues to individual identity and potentially to other attributes associated with that individual. Some of these novel features may prove to be more ‘successful’ than others: they may be more effective at signalling species, population, or individual identity, more flexible in signalling intention or motivation, or more informative about individual quality. Successful features may then be copied more often and increase within the population because of cultural selection. Random processes, or cultural drift, may also result in spreading novel song forms within a population. In these ways, successive rounds of vocal learning can build upon, change, elaborate, and increase the efficiency of song features, without calling on an evolutionary drive to produce an increased number of distinguishable signals.

## 7. Conclusion

Reduced to their most basic building blocks, bird songs consist of a set of notes. General cognitive functions such as species-specific sensory predispositions, memorization and simple learning biases also serve as building blocks, working together to group the notes into categories during social learning. A third type of building block, sequencing, forms simple strings, or syllables, that include notes from two or more categories. In the case of swamp sparrows, a bird may produce several syllable types; each song consists of a single syllable type repeated several times. In other species, different syllable types are concatenated, forming a learned sequence with two levels of structure—an elaboration that increases the complexity of the song. Different song segments may then diverge in function over time, with different pressures defining how each segment evolves, increasing the efficacy of each segment in terms of the communication role it plays. Although bird songs do not, as far as we know, have combinatorial structure that can be used to generate referential meaning, they do meet the criteria for cumulative cultural evolution: the songs are socially learned, a population's songs change systematically over time, and a comparative approach reveals that structures—the note categories and rules for assembling notes into syllables and syllables into songs—are elaborated in ways that increase complexity. In songs such as those of the Savannah sparrow, different segments carry different information, and cultural evolution of those song segments increases their efficiency. Although bird songs lack the evolutionary pressure for combinatorial structure that referential meaning engenders, simple building blocks (acoustically variable notes and general cognitive mechanisms) are sufficient to give rise to vocal signals with enough attributes for social learning and evolutionary mechanisms to incrementally produce increased complexity and efficacy—the hallmarks of cumulative cultural evolution.

## Endnote

1 Although terminology differs by author and species, in general ‘notes’ are continuous sound elements, ‘syllables’ are clusters of notes separated by short (less than 10 ms) silent intervals delivered in a stereotyped order and demarcated by being repeated or having longer (but consistent) silent intervals at the beginning and end of the syllable than within the syllable, ‘phrases’ are groups of syllables that are sung in a consistent (sometimes repeated) order within a song and may be demarcated by slightly longer (but again consistent) silent intervals at the boundaries of the phrase, and ‘songs’ are groups of syllables or phrases separated by longer (greater than 0.5 s) and more variable silent intervals (see examples in figures 2 and 5). ‘Bouts’ are groups of songs that are separated by still longer and more variable silent intervals.

## Data accessibility

This article has no additional data.
